# Stimulation of Transient Receptor Potential Channels TRPM3 and TRPM8 Increases Human Prostaglandin Endoperoxide Synthase-2 Promoter Activity

**DOI:** 10.3390/molecules30163320

**Published:** 2025-08-08

**Authors:** Nikolas Brandmeier, Oliver G. Rössler, Gerald Thiel

**Affiliations:** Department of Medical Biochemistry and Molecular Biology, Medical Faculty, University of Saarland, Campus Homburg, 66421 Homburg, Germany; brandmeiernikolas@gmail.com (N.B.); oliver.roessler@uks.eu (O.G.R.)

**Keywords:** TRPM3, TRPM8, human prostaglandin endoperoxide synthase-2, cAMP response element, CREB, mefenamic acid, RQ-00203078

## Abstract

The transient receptor potential channels TRPM3 and TRPM8 are cation channels that regulate numerous cellular activities, including thermo- and pain sensation. Stimulation of either TRPM3 or TRPM8 channels induces an intracellular signaling cascade that leads to the activation of stimulus-responsive transcription factors. As part of a search for delayed-response genes that are activated upon TRPM3 or TRPM8 stimulation, we analyzed the gene encoding prostaglandin endoperoxide synthase-2. The expression of this gene is not detectable under basal conditions but is rapidly induced upon stimulation of the cells with numerous extracellular signaling molecules. Here, we show that chromatin-embedded reporter genes under the control of the prostaglandin endoperoxide synthase-2 promoter were activated after stimulation of TRPM3 channels with pregnenolone sulfate or TRPM8 channels with the cooling agent icilin. TRP channel-induced activation of the prostaglandin endoperoxide synthase-2 promoter was attenuated by pharmacological inhibitors of TRPM3 and TRPM8. Mutational analysis of the prostaglandin endoperoxide synthase-2 promoter showed the importance of a cAMP response element within the proximal promoter region of the prostaglandin endoperoxide synthase-2 gene. In summary, our results establish a link between the stimulation of TRPM3 and TRPM8 and the biosynthesis of proinflammatory mediators via the regulation of prostaglandin endoperoxide synthase-2 expression.

## 1. Introduction

Transient-receptor potential (TRP) channels are non-selective cation channels that have numerous biological functions, including sensing temperature and pain, regulating secretion, and neurotransmission [1]. TRP channels are considered promising targets for the treatment of human diseases [2].

TRPM3 channels are involved in temperature and pain sensation, the regulation of peptide secretion, muscle contraction, gene transcription, and tumorigenesis [3,4]. Analysis of transgenic mice models revealed an important role of TRPM3 in heat sensation and the development of inflammatory heat sensitization [5,6]. TRPM3 channels have been proposed as molecular markers for chronic fatigue syndrome/myalgic encephalomyelitis [7]. Furthermore, analysis of mutated TRPM3 channels revealed that TRPM3 plays an important role in the development of neuronal disorders, including epileptic encephalopathies [8] and the development of an inherited form of early-onset cataract [9]. TRPM8 was originally identified as the menthol receptor in an expression cloning approach [10]. TRPM8 is activated by cold temperatures and plays an important role in thermosensation [11,12,13]. Other functions of TRPM8 include the development of migraine and tumorigenesis [14,15].

Stimulation of TRPM3 or TRPM8 channels induces an intracellular signaling pathway that leads to a change in the gene expression pattern of the cells via the activation of stimulus-responsive transcription factors [3,16,17]. These transcription factors bind to the regulatory region of delayed-response genes and stimulate transcription of these genes. The gene products of these delayed-response genes are then responsible for the biochemical and physiological changes observed after cellular stimulation. Identification of these genes will contribute to a better understanding of the biological functions of these TRP channels. We recently identified the gene encoding the proinflammatory cytokine interleukin-8 (IL-8), also known as CXCL8, as a delayed-response gene to TRPM3 stimulation [18]. IL-8 acts as a chemoattractant and a neutrophil activator, and the expression and secretion of IL-8 have been associated with inflammatory disorders. It has also been shown that stimulation of TRPM3 channels induces the secretion of calcitonin gene-related peptide (CGRP) in skin nerve terminals stimulated with pregnenolone sulfate [19]. CGRP is a neuropeptide synthesized and released by nociceptors and acts as a neuromodulator inducing inflammation and pain.

Prostaglandins act as mediators of an inflammatory response. They are synthesized from arachidonic acid in a reaction catalyzed by prostaglandin endoperoxide synthases 1 and 2, also known as cyclooxygenases 1 and 2. In a search for additional delayed-response genes of TRPM3- and TRPM8-induced signaling, we analyzed the gene encoding prostaglandin endoperoxide synthase-2 (EC 1.14.99.1). The enzyme catalyzes the rate-limiting reaction in prostaglandin biosynthesis and is therefore a therapeutic target for anti-inflammatory medications, e.g., non-steroidal anti-inflammatory drugs. Prostaglandin endoperoxide synthase-2 is expressed at very low levels in various tissues but can be rapidly induced by inflammatory stimuli such as inflammatory cytokines and lipopolysaccharides as well as numerous other signaling molecules, e.g., tumor promoters and hormones [20]. In contrast, the prostaglandin endoperoxide synthase-1 isoenzyme is constitutively expressed.

Here, we analyzed the promoter of prostaglandin endoperoxide synthase-2 after stimulation of either TRPM3 or TRPM8 channels. We show that the activity of the prostaglandin endoperoxide synthase-2 promoter increases after stimulation of TRPM3 or TRPM8 channels with their cognate ligands. We identified the cAMP response element (CRE) as an important genetic landmark that links TRPM3 and TRPM8 channel stimulation with the activation of the prostaglandin endoperoxide synthase-2 gene promoter. Overall, this is the first report linking TRPM3 and TRPM8 channel stimulation to the regulation of the prostaglandin endoperoxide synthase-2 gene.

## 2. Results

### 2.1. Prostaglandin Endoperoxide Synthase-2 Catalyzes the Biosynthesis of Prostaglandin H_2_ (PGH_2_)

**Figure 1:**
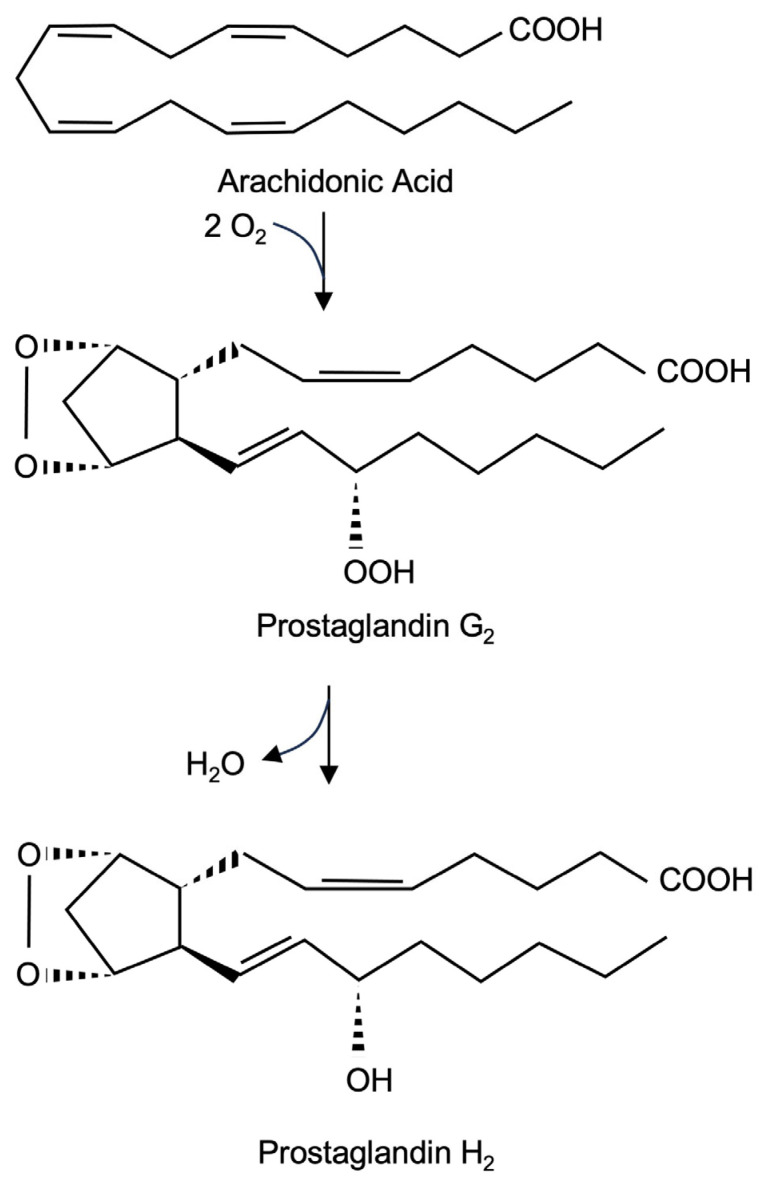
Biosynthesis of prostaglandin PGH_2_ from arachidonate. The isoenzymes prostaglandin endoperoxide synthases 1 and 2 catalyze the reaction in two steps, with prostaglandin PGG_2_ serving as an intermediate.

Prostaglandin endoperoxide synthase-2 is a bifunctional enzyme (Figure 1). First, it catalyzes the conversion of arachidonate into hydroperoxide prostaglandin G_2_ (PGG_2_) using its cyclooxygenase activity, which results in the incorporation of two molecules of O_2_ into arachidonic acid. Second, the enzyme uses its hydroperoxide activity to catalyze a two-electron reduction in the hydroperoxide moiety of PGG_2_ to alcohol, resulting in the formation of PGH_2_. There are two prostaglandin endoperoxide synthase isoenzymes. Prostaglandin endoperoxide synthase-1 is constitutively expressed, while the expression of prostaglandin endoperoxide synthase-2 is induced by a variety of extracellular signaling molecules. The biological activity of prostaglandin endoperoxide synthase-2 to catalyze the biosynthesis of prostaglandins is associated with the induction of inflammation, pain, and fever. It is therefore obvious that research is trying to clarify how the expression of the enzyme is regulated and how the enzyme can be pharmacologically inhibited.

### 2.2. TRPM3 Channel Stimulation Activates the Prostaglandin Endoperoxide Synthase-2 Promoter

**Figure 2:**
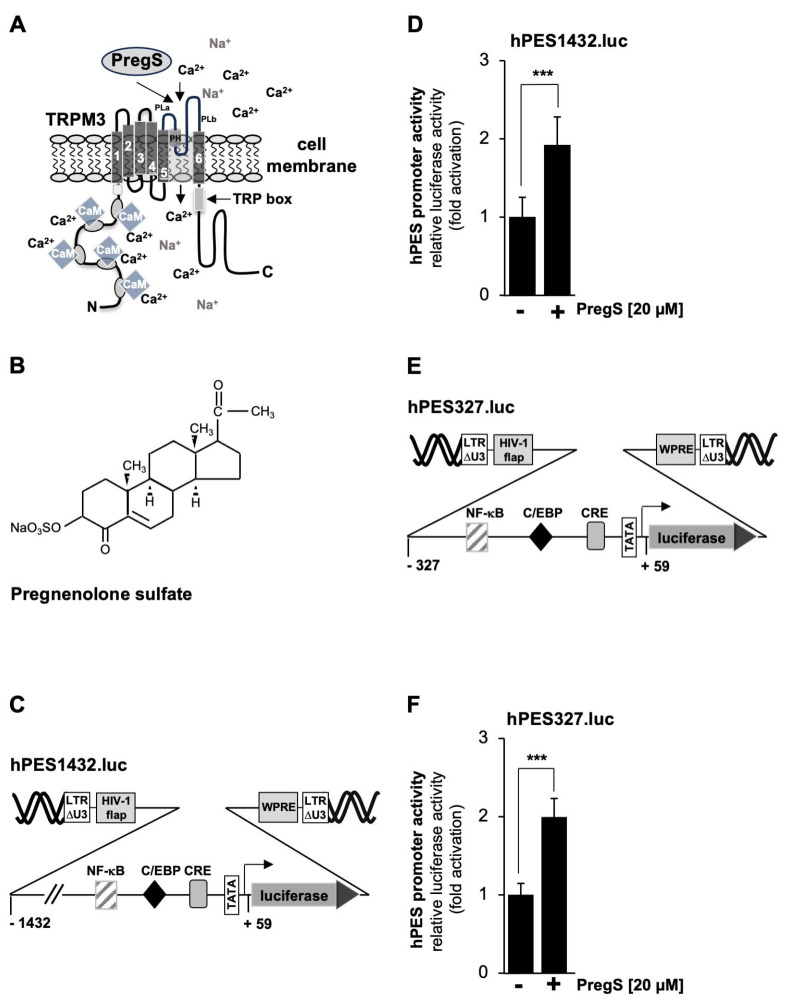
Stimulation of TRPM3 channels with pregnenolone sulfate increases prostaglandin endoperoxide synthase-2 promoter activity. (**A**) Domain structure of TRPM3 channels. TRPM3 channels are homotetramers centered around a central pore. It has been suggested that pregnenolone sulfate directly interacts with the pore helix [21]. Numerous putative calmodulin (CaM) binding sites have been identified in the N-terminal cytoplasmic region. The TRP box, along with other domains, is involved in the binding of phosphatidylinositol 4,5-bisphosphate. (**B**) Chemical structure of pregnenolone sulfate. (**C**) Schematic representation of the provirus containing the luciferase reporter gene under the control of 1432 nucleotides of the 5’upstream region of the human prostaglandin endoperoxide synthase-2 gene. (**D**) HEK293 cells containing a tetracycline-inducible TRPM3 transcription unit were infected with a recombinant lentivirus containing a prostaglandin endoperoxide synthase-2 promoter/luciferase reporter gene (hPES1432.luc). The cells were serum-starved for 24 h in the presence of tetracycline (1 μg/mL) and then stimulated with pregnenolone sulfate (PregS, 20 μM) for 24 h. Cell extracts were prepared and analyzed for luciferase activities and protein concentrations. The luciferase activities were normalized to the protein concentrations of the extracts. The data shown are the means ± SDs of six experiments performed in quadruplicate (*** *p* < 0.001). (**E**) Schematic representation of the provirus, which contained the luciferase reporter gene under the control of 327 nucleotides of the 5’upstream region of the human prostaglandin endoperoxide synthase-2 gene. (**F**) HEK293 cells containing a tetracycline-inducible TRPM3 transcription unit were infected with a recombinant lentivirus containing a prostaglandin endoperoxide synthase-2 promoter/luciferase reporter gene (hPES327.luc). Cells were serum-starved for 24 h in the presence of tetracycline (1 μg/mL) and then stimulated with pregnenolone sulfate (20 μM) for 24 h. Data shown are the means ± SDs of three experiments performed in quadruplicate (*** *p* < 0.001).

As a cellular model system to analyze TRPM3 signaling, we used HEK293 cells that contained a tetracycline-inducible TRPM3 expression unit (T-REx-TRPM3 cells). TRPM3 expression could therefore be induced by adding tetracycline to the culture medium. Figure 2A shows the modular structure of TRPM3 channels and reveals the architecture common to all TRP channels, including six transmembrane domains and a cytoplasmic location of both N- and C-termini. The ion pore of TRPM3 involves the loop between the fifth and sixth transmembrane domains. TRPM3 channels are activated by pregnenolone sulfate (PregS) (Figure 2B), resulting in an influx of Ca^2+^ or Na^+^ ions into the cytosol of the cells. It has been suggested that pregnenolone sulfate binds to the pore region of the channel [21].

Stimulation of TRPM3 channels results in the transient activation of stimulus-responsive transcription factors, which in turn bind to the regulatory region of delayed-response genes. The expression of these delayed-response genes is responsible for altering the biochemistry of the cells due to TRPM3 channel stimulation. We wondered whether the prostaglandin endoperoxide synthase-2 gene is one of the TRPM3-activated delayed- response genes, since the expression of this gene is regulated by numerous extracellular signaling molecules. Furthermore, it has been demonstrated that an increase in the intracellular Ca^2+^ ion concentration and activation of PKC and extracellular signal-regulated protein kinase ERK1/2 activate the promoter of the prostaglandin endoperoxide synthase-2 gene [22,23,24,25,26,27,28,29,30,31]. An increase in the intracellular Ca^2+^ ion concentration and the activation of PKC and ERK1/2 are also characteristics of the TRPM3-induced signaling cascade [32,33,34].

We used lentiviral gene transfer to integrate the reporter gene into the genome of the cells. Thus, the reporter gene was embedded into a nucleosomal context. Integration of reporter genes into the chromatin is important because transcriptional regulation requires not only the binding of transcription factors to DNA but also a nucleosomal structure typical for eukaryotic genes. The transient transfection of reporter genes used by many researchers leads to inefficiently packed plasmids into chromatin with incomplete nucleosomal organization [35,36]. Furthermore, these reporter genes have a prokaryotic-like gene organization with the hallmark of a non-restrictive transcriptional ground state, whereas eukaryotic genes are embedded into a chromatin-structured restrictive ground state. Implanting reporter genes into chromatin ensures that they are packed into an ordered nucleosomal structure.

Figure 2C shows a schematic representation of the integrated provirus encoding a luciferase reporter gene under the control of 1432 nucleotides of the 5’upstream region of the human prostaglandin endoperoxide synthase-2 gene. T-REx-TRPM3 cells were infected with a recombinant lentivirus containing the prostaglandin endoperoxide synthase-2 promoter/luciferase reporter gene, cultured in a serum-reduced medium in the presence of tetracycline to induce TRPM3 expression, and then stimulated with prognenolone sulfate for 24 h. Cells were harvested, and cell extracts were prepared and analyzed for luciferase activities and protein concentrations. The results show that stimulation of TRPM3 channels increased the promoter activity of the prostaglandin endoperoxide synthase-2 gene by 1.92-fold (Figure 2D). The proximal prostaglandin endoperoxide synthase-2 promoter contains three landmark transcription factor binding sites, which were identified as binding sites for nuclear factor ‘kappa-light-chain-enhancer’ of activated B-cells (NF-κB), the CCAAT/enhancer-binding protein (C/EBP) (also termed nuclear factor for IL-6 expression, NF-IL6 [37]), and the cAMP response element-binding protein (CREB). We wondered whether the proximal promoter of the prostaglandin synthase-2 gene is sufficient to confer responsiveness to TRPM3 stimulation. We therefore analyzed a reporter gene under the control of 327 nucleotides of the prostaglandin endoperoxide synthase-2 promoter (Figure 2E). The results show that TRPM3 stimulation triggered a 1.99-fold upregulation of prostaglandin endoperoxide synthase-2 promoter activity (Figure 2F), suggesting that the genetic elements required for stimulus–transcription coupling are located in the proximal promoter region.

### 2.3. Pharmacological Inhibition of TRPM3 Channels Impairs Pregnenolone Sulfate-Induced Upregulation of Prostaglandin Endoperoxide Synthase-2 Promoter Activity

**Figure 3:**
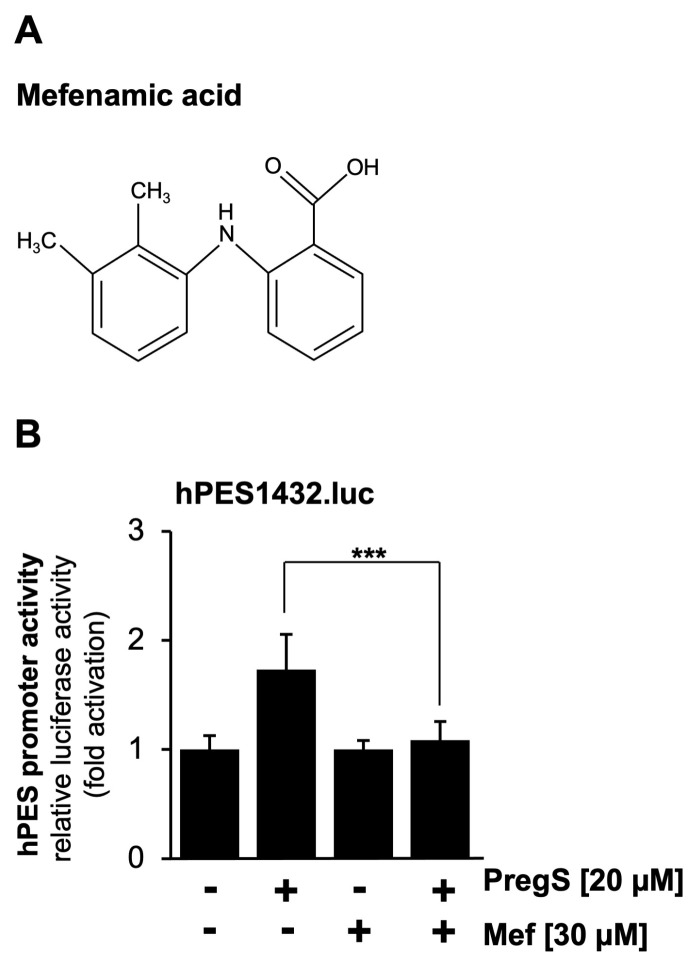
Mefenamic acid inhibits pregnenolone sulfate-induced stimulation of the prostaglandin endoperoxide synthase-2 promoter in TRPM3-expressing HEK293 cells. (**A**) Chemical structure of mefenamic acid (Mef). (**B**) HEK293 cells containing a tetracycline-inducible TRPM3 transcription unit were infected with a recombinant lentivirus containing the prostaglandin endoperoxide synthase-2 promoter/luciferase reporter gene (hPES1432.luc). The cells were serum-starved for 24 h in the presence of tetracycline (1 μg/mL) and then stimulated with pregnenolone sulfate (PregS, 20 μM) in the presence or absence of mefenamic acid (30 μM) for 24 h. Cell extracts were prepared and analyzed for luciferase activities and protein concentrations. Luciferase activity was normalized to the protein concentration. Data shown are the means ± SDs of three experiments performed in quadruplicate (*** *p* < 0.001).

We used a pharmacological approach to confirm that pregnenolone sulfate-induced activation of the prostaglandin endoperoxide synthase-2 promoter is dependent on TRPM3. We incubated the cells with mefenamic acid, an anthranilic acid derivative of non-steroid anti-inflammatory drugs, which selectively inhibits TRPM3-mediated Ca^2+^ influx into the cells and TRPM3-induced gene transcription [38,39,40]. The chemical structure of mefenamic acid is shown in Figure 3A. The results show that incubation of the cells with mefenamic acid greatly reduced pregnenolone sulfate-induced stimulation of the prostaglandin endoperoxide synthase-2 promoter (Figure 3B).

### 2.4. Mutational Analysis of the Human Prostaglandin Endoperoxide Synthase-2 Promoter Identified the CRE as the Pregnenolone Sulfate-Responsive Element

**Figure 4:**
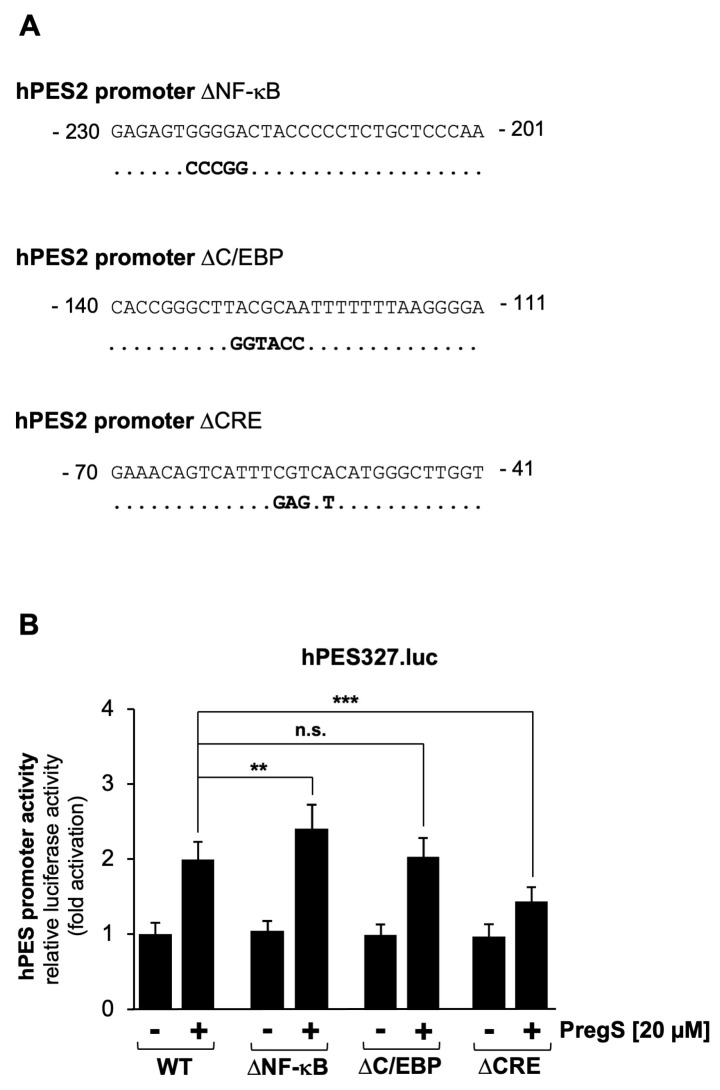
Mutational analysis identifies the CRE as the pregnenolone sulfate-responsive element within the prostaglandin endoperoxide synthase-2 promoter. (**A**) Sequence of the human prostaglandin endoperoxide synthase-2 promoter showing mutated base pairs within the binding sites for NF-κB and C/EBP as well as within the CRE. (**B**) HEK293 cells containing a tetracycline-inducible TRPM3 transcription unit were infected with recombinant lentiviruses containing a luciferase reporter gene under the wild-type proximal prostaglandin endoperoxide synthase-2 promoter (hPES327.luc) or three mutated versions of the prostaglandin endoperoxide synthase-2 promoter (ΔNF-κB, ΔC/EBP, ΔCRE) The cells were serum-starved for 24 h in the presence of tetracycline (1 μg/mL) and then stimulated with pregnenolone sulfate (PregS, 20 μM) for 24 h. Cell extracts were prepared and analyzed for luciferase activities. Luciferase activity was normalized to the protein concentration. Data shown are the mean ± SD from three independent experiments, each performed in quadruplicate (** *p* < 0.01, *** *p* < 0.001, n.s.: not significant).

The proximal promoter of the human prostaglandin endoperoxide synthase-2 gene contains three key genetic elements: binding sites for the transcription factors NF-κB and C/EBP and a CRE, which is a binding site for the transcription factor CREB and related basic-region leucine zipper transcription factors. Mutations were introduced into these elements, as shown in Figure 4A, and they were analyzed for their responsiveness to pregnenolone sulfate. Figure 4B shows that only mutations introduced into the CRE impaired pregnenolone sulfate-induced activation of the prostaglandin endoperoxide synthase-2 promoter in HEK293 cells expressing TRPM3 channels. Transcription of the reporter gene was reduced by 50%. Mutations introduced into the NF-κB and C/EBP binding sites did not reduce the responsiveness of the prostaglandin endoperoxide synthase-2 promoter to the TRPM3-induced signaling cascade. Mutation of the NF-κB binding site surprisingly increased prostaglandin endoperoxide synthase-2 promoter activity.

### 2.5. TRPM8 Channel Stimulation Activates the Prostaglandin Endoperoxide Synthase-2 Promoter

**Figure 5:**
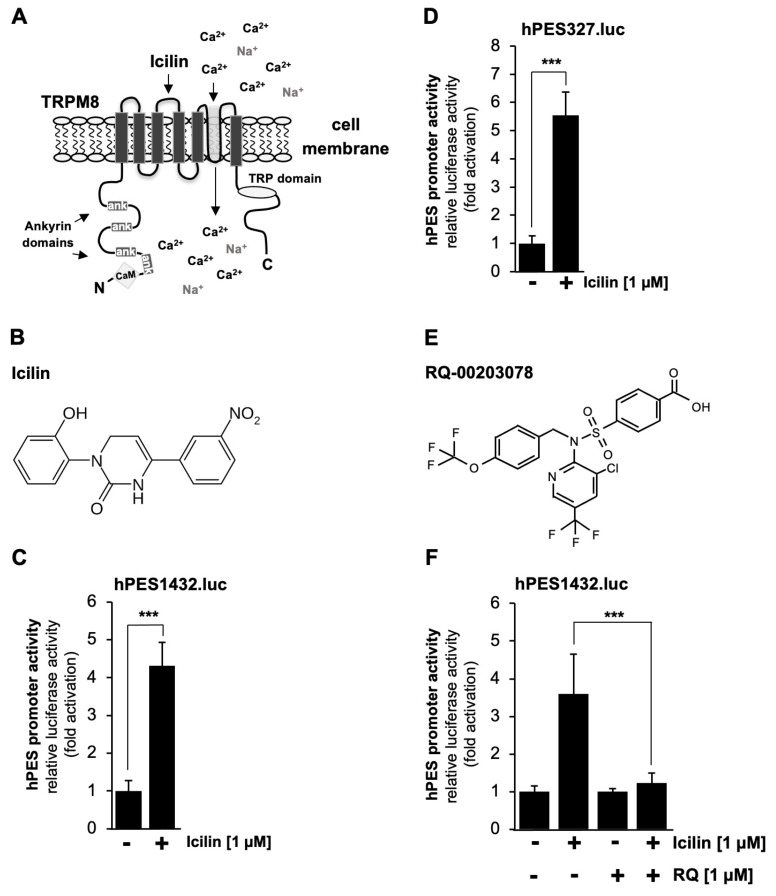
Stimulation of TRPM8 channels with icilin increases the promoter activity of the prostaglandin endoperoxide synthase-2 gene. (**A**) Domain structure of TRPM8 channels, showing the typical transmembrane organization of TRP channels. Ankyrin domains, a TRP box, and a putative calmodulin (CaM) binding site are shown. (**B**) Chemical structure of icilin, a highly potent agonist of TRPM8 channels. (**C**,**D**) HEK293-M8 cells, HEK293 cells expressing TRPM8 channels, were infected with lentiviruses containing a luciferase reporter under the control of either 1432 (**C**) or 327 nucleotides (**D**) of the 5′-upstream region of the prostaglandin endoperoxide synthase-2 promoter. Cells were serum-starved for 24 h and then stimulated with icilin (1 μM) for 24 h. Cells were harvested and analyzed as described in the legend in Figure 2. Data shown are the means ± SDs of six (**C**) or four (**D**) experiments performed in quadruplicate (*** *p* < 0.001). (**E**) Chemical structure of RQ-00203078. (**F**) HEK293-M8 cells were infected with a recombinant lentivirus containing the prostaglandin endoperoxide synthase-2 promoter/luciferase reporter gene (hPES1432.luc). Cells were serum-starved for 24 h, preincubated for 3 h with the compound RQ-00203078 (1 μM), and then stimulated with icilin (1 μM) in the presence or absence of RQ-00203078 (1 μM) for 24 h. Cell extracts were prepared and analyzed for luciferase activities and protein concentrations. Luciferase activity was normalized to the protein concentration. Data shown are the means ± SDs of four experiments, which were performed in quadruplicate (*** *p* < 0.001).

Next, we analyzed whether the prostaglandin endoperoxide synthase-2 gene could also function as delayed-response gene of the signaling pathway induced after stimulation of TRPM8 channels, cation channels permeable for Ca^2+^ and Na^+^ ions [41]. TRPM8 has the typical architecture of TRP channels with six transmembrane domains and both N- and C-termini in the cytoplasm as shown in Figure 5A. The figure also shows the TRP box, ankyrin repeats. and putative calmodulin binding sites. TRPM8 channels are activated following incubation of the cells with plant-derived “cooling agents” such as menthol or eucalyptol. In our experiments, we used the synthetic super-cooling agent icilin to stimulate TRPM8 (Figure 5B). The results show that icilin significantly induced transcription of the reporter gene under the control of either 1432 (Figure 5C) or 327 nucleotides (Figure 5D) of the 5’region of the human prostaglandin endoperoxide synthase-2 gene. The stimulation was in the range of 4.3-fold (hPES1432.luc) and 5.55-fold (hPES327.luc), respectively. These data also show that the genetic elements responsible for coupling TRPM8 stimulation with prostaglandin endoperoxide synthase-2 gene activation reside in the proximal portion of the prostaglandin endoperoxide synthase-2 promoter.

### 2.6. Pharmacological Inhibition of TRPM8 Channels Impairs Icilin-Induced Upregulation of the Prostaglandin Endoperoxide Synthase-2 Promoter

Recently, we showed that the compound RQ-00203078 specifically inhibits TRPM8-mediated transcriptional regulation [42]. This compound, shown in Figure 5E, also inhibits menthol-induced Ca^2+^ influx and whole-cell current as well as icilin-induced rise in the intracellular Ca^2+^ concentration in TRPM8-expressing HEK293 cells [43,44] (https://www.alomone.com/p/rq-00203078/R-170?b=33803 (accessed on 8 July 2025)). We therefore used this compound to confirm that icilin-induced upregulation of r eporter gene transcription relies on the presence of functional TRPM8 channels. The results show that incubation of the cells with RQ-00203078 almost completely blocked transcriptional activation of the prostaglandin endoperoxide synthase-2 promoter in icilin-stimulated HEK293-M8 cells (Figure 5F). The stimulation of reporter gene transcription was inhibited by more than 91%.

### 2.7. Mutational Analysis of the Human Prostaglandin Endoperoxide Synthase-2 Promoter Identified Multiple Genetic Elements as Icilin-Responsive Elements

**Figure 6:**
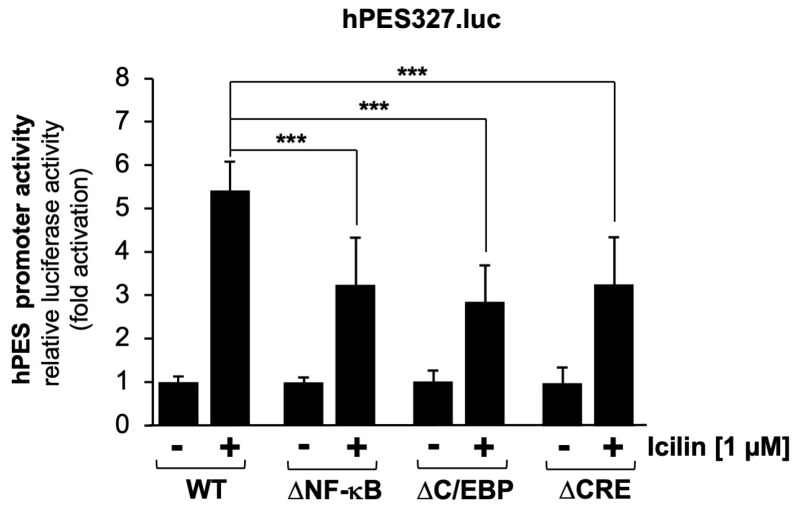
Mutational analysis identifies multiple icilin-responsive elements within the prostaglandin endoperoxide synthase-2 promoter. HEK293-M8 cells were infected with recombinant lentiviruses containing a luciferase reporter gene under the wild-type proximal prostaglandin endoperoxide synthase-2 promoter (hPES327.luc) or the mutant versions of the prostaglandin endoperoxide synthase-2 promoter (ΔNF-κB, ΔC/EBP, and ΔCRE). Cells were serum-starved for 24 h and then stimulated with icilin (1 μM) for 24 h. Cell extracts were prepared and analyzed for luciferase activities. Luciferase activity was normalized to the protein concentration. Data shown are the mean ± SD from three independent experiments, which were carried out in quadruplicate (*** *p* < 0.001).

Next, we analyzed mutants of the prostaglandin endoperoxide synthase-2 promoter. Figure 6 shows that mutations of the NF-κB and C/EBP binding sites, as well as mutations introduced into the CRE, impaired the activation of the prostaglandin endoperoxide synthase-2 promoter by icilin. Quantification revealed a reduction in reporter gene transcription of the order of 48.9% (ΔNF-κB), 58.2% (ΔC/EBP), and 48.6% (ΔCRE), respectively.

### 2.8. TRPM8 Channel Stimulation Activates a CRE-Containing Reporter Gene

**Figure 7:**
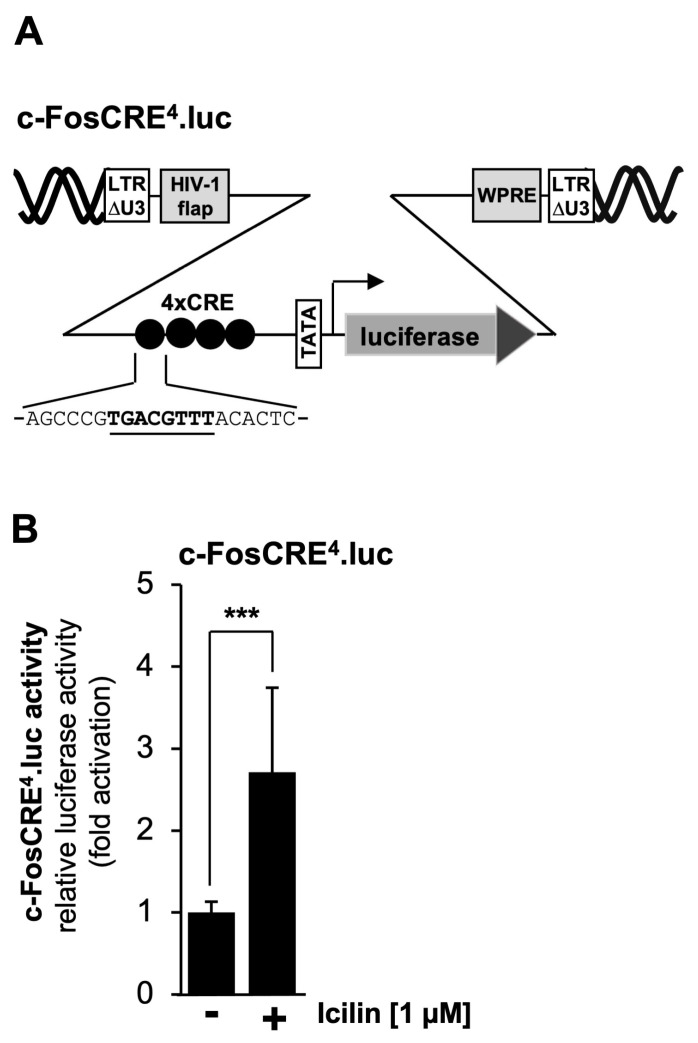
Transcriptional response of a CRE-containing promoter to TRPM8 stimulation. (**A**) Schematic representation of a provirus containing the luciferase reporter gene under the control of 4 CREs derived from the c-Fos gene. (**B**) HEK293-M8 cells were infected with a recombinant lentivirus generated with the lentiviral transfer vector pFWc-FosCRE^4^.luc. The cells were serum-starved for 24 h and then stimulated with icilin (1 μM) for 24 h. Cell extracts were prepared and analyzed for luciferase activities. Luciferase activity was normalized to the protein concentration. Data shown are the mean ± SD from three independent experiments performed in quadruplicate (*** *p* < 0.001).

Mutational analysis of the prostaglandin endoperoxide synthase-2 promoter revealed that the CRE is important for TRPM3 and TRPM8 signaling. It has already been shown that stimulation of TRPM3 channels activates a reporter gene under the control of four copies of the canonical CRE sequence 5′-TGACGTCA-3′ [45,46,47], as depicted in Figure 7A. Figure 7B shows that stimulation of TRPM8 channels with icilin also activated transcription of a reporter gene controlled by four CREs. Transcription was stimulated by a factor of 2.71.

### 2.9. Activation of CRE-Containing Genes Following Stimulation of TRPM3 or TRPM8 Channels

**Figure 8:**
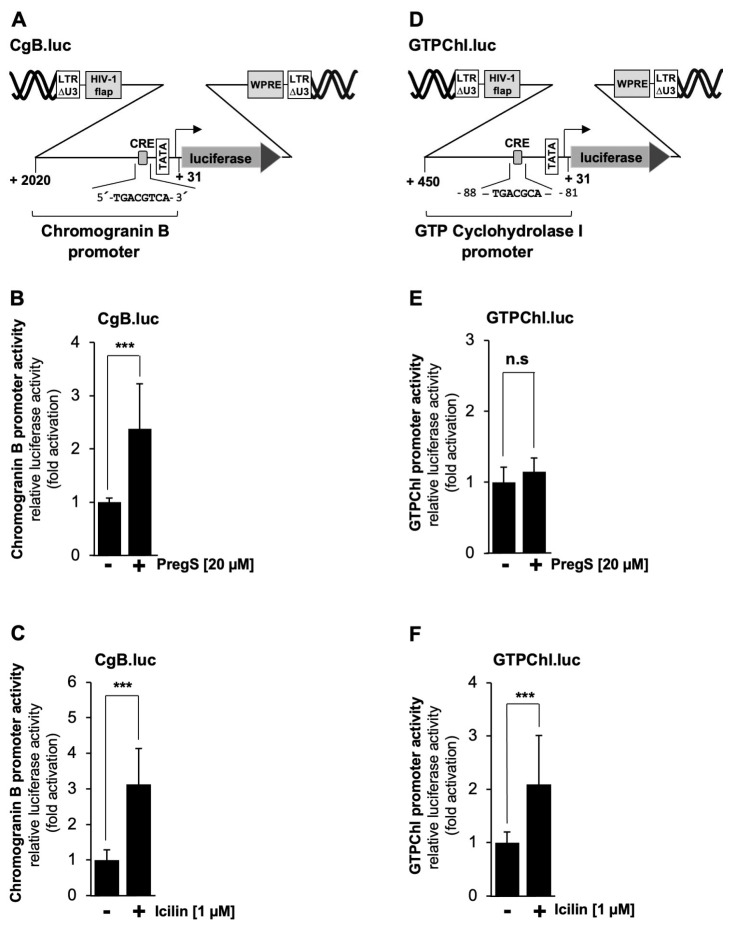
Transcriptional response of CRE-containing promoters to TRPM3 or TRPM8 stimulation. (**A**) Schematic representation of a provirus containing the luciferase reporter gene under the control of the human chromogranin B (CgB) promoter. The location of the canonical CRE is indicated. (**B**,**C**) T-REx-TRPM3 cells (**B**) and HEK293-M8 cells (**C**) were infected with a lentivirus containing the chromogranin B promoter/luciferase reporter gene. Cells were stimulated with pregnenolone sulfate or icilin, harvested, and processed as described in the legends in Figure 2. Data shown are the mean ± SD from three (**B**) or four (**C**) independent experiments, each performed in quadruplicate (*** *p* < 0.001). (**D**) Schematic representation of a provirus containing the luciferase reporter gene under the control of the human GTP cyclohydrolase I promoter. The location of the CRE-like sequence is indicated. (**E**,**F**) T-REx-TRPM3 cells (**E**) and HEK293-M8 cells (**F**) were infected with a lentivirus containing the GTP cyclohydrolase I promoter/luciferase reporter gene. Cells were stimulated with pregnenolone sulfate or icilin, harvested, and processed as described in the legends in Figure 2. The data presented are the mean ± SD from three independent experiments, which were performed in quadruplicate (*** *p* < 0.001; n.s., not significant).

Next, we analyzed the chromogranin B promoter, which contains one copy of a canonical CRE (Figure 8A). It has been demonstrated that transcription of the chromogranin B gene is regulated by this element [48]. Figure 8B and 8C show that stimulation of TRPM3 or TRPM8 channels induced transcription of the reporter gene under the control of the chromogranin B promoter by 2.4-fold and 3.1-fold, respectively. The promoter of the GTP cyclohydrolase I gene contains an atypical version of the CRE, encompassing the sequence 5′-TGACGCAA-3′ (Figure 8D). Nevertheless, this promoter was shown to respond to a constitutively active mutant of CREB [49]. Figure 8E shows that stimulation of TRPM3 channels had no effect on the promoter activity of this gene, while stimulation of TRPM8 channels activated a reporter gene controlled by the GTP cyclohydrolase I promoter by a factor of 2.1 (Figure 8F).

## 3. Discussion

Stimulation of TRPM3 and TRPM8 channels induces a signaling cascade that leads to the activation of the stimulus-responsive transcription factors AP-1, CREB, Elk-1, and Egr-1. AP-1 is a transcription factor complex composed of two basic-region leucine zipper transcription factors. AP-1 regulates numerous biological functions in the cells, including the regulation of proliferation, differentiation, and cell death [50]. Egr-1 is a zinc finger transcription factor involved in the regulation of cell proliferation [51,52]. Elk-1 functions as a master regulator of stimulus-induced gene transcription, regulating cellular proliferation, programmed cell death, glucose homeostasis, and brain function [53]. These proteins bind to the regulatory regions of delayed-response genes and activate transcription of these genes. To understand the intracellular functions of TRPM3 and TRPM8, the identification of these delayed-response genes is essential. Previous studies identified the genes encoding IL-8 and CGRP as delayed-response genes that are activated following stimulation of TRPM3 channels. Both peptides induce a proinflammatory response and thus provide a link between TRP channel activation and inflammation.

In the search for delayed-response genes activated following stimulation of TRPM3 or TRPM8 channels, we analyzed the gene encoding prostaglandin endoperoxide synthase-2. This enzyme is responsible for the biosynthesis of prostaglandins, which are important mediators of an inflammatory response. Under basal conditions, the expression of prostaglandin endoperoxide synthase-2 is undetectable in many tissues. However, stimulation of the cells with cytokines, growth factors, or tumor promoters triggers a strong transcriptional response, frequently involving PKC and the extracellular signal-regulated protein kinase ERK1/2 [23,24,25,27,29,30,31]. An increase in intracellular Ca^2+^ has also been shown to stimulate prostaglandin endoperoxide synthase-2 gene expression [25,28,31]. Since the TRPM3-induced intracellular signaling pathway has been shown to include an increase in the intracellular Ca^2+^ concentration, as well as the activation of PKC and ERK1/2 [32,34,54], we hypothesized that the prostaglandin endoperoxide synthase-2 promoter would respond to TRPM3 and TRPM8 stimulation. The experiments shown in this study identified the prostaglandin endoperoxide synthase-2 gene as a delayed-response gene of the pregnenolone sulfate/TRPM3 and the icilin/TRPM8-induced signaling pathways. The essential role of both channels in stimulus–transcription coupling was confirmed in experiments with specific inhibitors against TRPM3 and TRPM8.

The activation of TRPM3 channels is associated with heat sensation and the development of inflammatory heat hyperalgesia in the somatosensory system. Stimulation of TRPM3 channels has also been linked to tumorigenesis. TRPM8 channels are involved in cold hypersensitivity triggered by nerve injury and inflammation. TRPM8 channels are also thought to be involved in the development of tumors. Chronic inflammation may be involved in tumorigenesis. Prostaglandins are proinflammatory mediators that may also be involved in the transition from acute to chronic inflammation. Inhibitors of prostaglandin endoperoxide synthase-2 act therefore as anti-inflammatory compounds, e.g., non-steroidal anti-inflammatory drugs. Stimulation of prostaglandin endoperoxide synthase-2 during inflammation can also trigger hyperalgesia, an increased sensitivity to pain, which leads to increased biosynthesis of prostaglandins. In this scenario, stimulation of TRPM3 and TRPM8 channels would intensify the inflammatory response, while treatment with TRPM3 and TRPM8 channel antagonists would have an anti-inflammatory effect. It has recently been shown that local administration of non-steroidal anti-inflammatory drugs reduced thermal and mechanical hyperalgesia after stimulation of the TRP channels TRPA1 or TRPV1 [55], underscoring the link between TRP channel stimulation, prostaglandin biosynthesis, hyperalgesia, and inflammation.

At the level of gene regulation, we have shown in this study that the CRE within the proximal prostaglandin endoperoxide synthase-2 promoter was important for the coupling of TRPM channel activation with the prostaglandin endoperoxide synthase-2 promoter. The CRE has also been identified as essential for ultraviolet B irradiation-induced transcription of the prostaglandin endoperoxide synthase-2 gene [56]. Likewise, the upregulation of prostaglandin endoperoxide synthase-2 gene transcription by nitric oxide or platelet-derived growth factor stimulation requires the CRE to act as cis-acting element [57,58].

Several transcription factors have been proposed to bind to the CRE of the prostaglandin endoperoxide synthase-2 promoter. While the canonical CRE serves as a docking site for the transcription factor CREB and the related protein activating transcription factor-1 (ATF-1), non-canonical CREs found in the prostaglandin endoperoxide synthase-2 promoter and the GTP cyclohydrolase I promoter can also bind additional basic-region leucin zipper transcription factors. The GTP cyclohydrolase I promoter, for instance, was shown to be activated by constitutively active mutants of CREB, ATF-2, and c-Jun [49], but stimulation experiments with a cAMP analog showed that it is not CREB but rather the basic-region leucine zipper transcription factors ATF-4 and C/EBPβ, members of the C/EBP family of basic-region leucine zipper transcription factors, which mediate the response to elevated cAMP [59]. Nevertheless, the GTP cyclohydrolase I promoter was not activated by the stimulation of TRPM3 channels, as shown in this study, suggesting that the GTP cyclohydrolase I promoter and the prostaglandin endoperoxide synthase 2 promoter are differently regulated. Regarding the prostaglandin endoperoxide synthase 2 promoter, the involvement of CREB and ATF-1 in the regulation of promoter activation induced by ultraviolet B radiation has been demonstrated [56]. Furthermore, in an analysis of transgenic mice expressing A-CREB, an inhibitor of CREB consisting of the leucine zipper domain of CREB coupled to an acid domain, it was shown that CREB is an important transcription factor for the expression of prostaglandin endoperoxide synthase-2 in a model of status epilepticus [60].

In contrast, other investigators have suggested that c-Jun interacts with the CRE of the prostaglandin endoperoxide synthase-2 promoter in epidermal growth factor-treated human epidermoid carcinoma cells [61]. CREB, ATF-2, and c-Jun stimulate transcriptional activation of the prostaglandin endoperoxide synthase-2 promoter via the CRE in nitric oxide-stimulated hypopharyngeal squamous cell carcinoma [58]. c-Jun was identified—in addition to CREB—as a transcription factor that is activated as a result of TRPM3 or TRPM8 stimulation [34,45,62]. For the signaling pathway induced by pregnenolone sulfate-induced stimulation of TRPM3 channels, other cis-acting elements of the prostaglandin endoperoxide synthase-2 promoter, such as the C/EBP and NF-κB binding sites, did not play a role in stimulus transcription coupling. This is consistent with previous results showing that TRPM3 signaling does not activate NF-κB-induced gene transcription [18]. The binding sites for NF-κB and C/EBP are important for the induction of the prostaglandin endoperoxide synthase-2 promoter after stimulation of the cells with lipopolysaccharides [62,63,64,65]. Likewise, both the NF-κB and C/EBP sites mediate prostaglandin endoperoxide synthase-2 promoter activation after stimulation of gonadotroph cells with gonadotropin-releasing hormone [28].

Analysis of the TRPM8-induced signaling pathway revealed that mutations within the C/EBP and NF-κB binding sites, as well as mutations within the CRE, reduced icilin-mediated stimulation of the prostaglandin endoperoxide synthase-2 promoter. It seems to be a common theme in the regulation of the prostaglandin endoperoxide synthase-2 gene that no single cis-acting element mediates stimulus–transcription coupling, but rather that two or three of the landmark cis-acting elements are required. For example, the binding sites for NF-κB, C/EBP, and the CRE are involved as cis-acting response elements for various stimuli, e.g., NO donors, phorbol esters, bradykinin, and interleukin-1β [58,66]. The C/EBP site and the CRE are essential for the stimulation of the prostaglandin endoperoxide synthase-2 promoter by a combined treatment with lipopolysaccharides and 12-*O*-tetradecanoylphorbol-13-acetate, while inactivation of only one of these cis-acting elements had little effect on promoter activity [63]. Similarly, transforming growth factor α-induced stimulation of the prostaglandin endoperoxide synthase-2 promoter requires the C/EBP site and the CRE [23].

## 4. Materials and Methods

### 4.1. Cell Culture and Reagents

T-REx-TRPM3 cells were kindly provided by David Beech and Yasser Majeed, University of Leeds, UK, and cultured as described [67]. TRPM3 expression was induced by adding tetracycline (1 μg/mL, Sigma-Aldrich GmbH, Taufkirchen, Germany, # T7680, dissolved in water) to the culture medium, which contained 0.05% fetal calf serum, for 24 h before stimulation with the TRPM3 ligand pregnenolone sulfate (PubChem CID: 105074). Stimulation with pregnenolone sulfate (20 μM, Sigma # P162, dissolved in DMSO) was carried out for 24 h in DMEM with 0.05% fetal bovine serum. Mefenamic acid (2- (2,3-dimethylphenyl)aminobenzoic acid, PubChem CID: 4044) was obtained from Santa Cruz, Heidelberg, Germany (No. sc-205380), dissolved in DMSO, and used at a concentration of 30 μM [40]. HEK293-M8 cells have been described elsewhere [68]. Cells were incubated in DMEM with 0.05% fetal calf serum for 24 h before stimulation with the cooling compound icilin (1 μM, Santa Cruz, Heidelberg, Germany, No. sc-201557). HEK293-M8 cells were incubated with the compound RQ-00203078 (PubChem CID: 49783953), which was dissolved in DMSO, at a concentration of 1 μM. The compound was a kind gift from Alomone labs, Jerusalem. T-REx-TRPM3 cells and HEK293-M8 cells were preincubated with their cognate inhibitors for 3 h and then stimulated in the presence of the inhibitors with either pregnenolone sulfate or icilin.

### 4.2. Lentiviral Infection

Viral particles were produced by triple transfection of HEK293-TN cells with packaging plasmid Δ8.91, encoding the viral proteins gag, pol, and rev; pCMV-G, which codes for the VSV envelope glycoprotein; and the transfer vector as described [69].

### 4.3. Reporter Assays

Reporter plasmids phPES2 (−1432/+59), phPES2 (−327/+59), KBM, ILM, and CRM, containing the luciferase ORF under the control of the human prostaglandin endoperoxide synthase-2 promoter reporter, were kindly provided by Hiroyasu Inoue, Department of Food Science and Nutrition, Faculty of Human Life and Environment, Nara Woman’s University, Nara, Japan [63,64,70]. Plasmids were cut with EcoRV and NheI, and the fragments were inserted into a lentiviral transfer vector upstream of the luciferase coding region. The plasmids were designated pFWhPES2.luc (−1432), pFWhPES2.luc (−327), pFWhPES2.lucDκB, pFWhPES2.lucDC/EBP, and pFWhPES2.lucDCRE, respectively. The plasmid p0.613hGCH1-GL3 was kindly provided by Gregory Kapatos, Wayne State University, Department of Psychiatry, Detroit, Michigan, USA [71]. To construct the lentiviral transfer vector pFWGTPChI.luc, we cloned a fragment of plasmid p0.613hGCH1-GL3, which comprised the sequences from −450 to +31 of the human GTP cyclohydrolase I gene, into a lentiviral transfer vector upstream of the luciferase coding region. The lentiviral transfer vectors pFWCgB.luc, containing the sequence −2020/+31 of the human chromogranin B promoter upstream of the luciferase ORF, and pFWCRE^4^.luc have already been described [48,72]. Infected cells were kept in a medium containing 0.05% fetal calf serum for 24 h before stimulation. Reporter lysis buffer (Promega, Mannheim, Germany) was used to prepare cell extracts, which were used to determine luciferase activities and protein concentrations. Luciferase activities were measured using a luminometer (Berthold Detection Systems, Huntsville, AL, USA). The protein concentrations of the extracts were determined using a BCA protein assay kit.

### 4.4. Statistics

For the statistical analyses, we used the two-tailed Student’s *t*-test. Statistical probability was expressed as *** *p* < 0.001. Values were considered significant if *p* < 0.05.

## 5. Conclusions

Previous studies showed that the genes encoding IL-8 and CGRP function as delayed-response genes that are activated following stimulation of TRPM3 channels. Both peptides induce a proinflammatory response and thus provide a link between TRP channel activation and inflammation. This study shows that the prostaglandin endoperoxide synthase-2 gene promoter is activated after stimulation of TRPM3 or TRPM8 channels, suggesting that stimulation of these TRP channels leads to the biosynthesis of the prostaglandin endoperoxide synthase-2 enzyme, which catalyzes the biosynthesis of prostaglandins, which are important mediators of an inflammatory response. In summary, the results of this study establish a link between the stimulation of TRPM3 or TRPM8 channels and the biosynthesis of proinflammatory mediators via the regulation of prostaglandin endoperoxide synthase-2. The fact that stimulation of TRPC6 and TRPM8 channels leads to an activation of IL-8 gene transcription, while stimulation of TRPV1 channels induces the biosynthesis of CGRP [18,73,74], suggests that other TRP channels also induce the biosynthesis of proinflammatory mediators. Next experiments should include the measurement of the enzyme activity of prostaglandin endoperoxide synthase-2, the concentration of prostaglandin peroxide synthase-2, and the concentration of prostaglandin H2 after stimulation of TRPM3 and TRPM8 channels. This requires experiments with primary cells that endogenously express TRPM3 or TRPM8 channels. The aim should be to confirm the link between TRPM3 and TRPM8 channel activity, the expression of prostaglandin endoperoxide synthase-2, and inflammation.

## Data Availability

Sequence ID: NG_028206.2 for homo sapiens prostaglandin endoperoxide synthase-2, RefSeqGene on chromosome 1.

## Abbreviations

The following abbreviations are used in this manuscript:

CRE	cyclic AMP response element
CREB	cyclic AMP response element-binding protein
ERK	extracellular signal-regulated protein kinase
PKC	protein kinase C
PregS	pregnenolone sulfate
TRP	transient receptor potential
